# Complete mitochondrial genome of the hybrid flounder *Paralichthys olivaceus* (♀) × *Verasper variegatus* (♂)

**DOI:** 10.1080/23802359.2025.2498746

**Published:** 2025-05-08

**Authors:** HyeJin Kim, YongHwi Kim, Bong Han Yun, Ho-Seop Han, Ho Sung Lee, In Gug Baek, In-Chul Bang

**Affiliations:** a Department of Biology, Soonchunhyang University, Asan, Republic of Korea; b Bio-laboratory, BioTNS Co., Ltd, Daejeon, Republic of Korea; c Institute of Korea Eco-Network, Daejeon, Republic of Korea

**Keywords:** F2, flatfish, hybridization, mitochondrial genome, phylogenetic, Pleuronectidae

## Abstract

Two generations were hatched after artificially crossing *Paralichthys olivaceus* and *Verasper variegatus*, commercially valuable species of Pleuronectidae. Here, we report the complete mitochondrial genome and phylogenetic relationships of *P. olivaceus* (♀) × *V. variegatus* (♂). The mitogenome is 16,946 bp long and contains 13 protein-coding genes (PCGs), 22 tRNA genes, and 2 rRNA genes. Phylogenetically, it clustered with *P. olivaceus* within Pleuronectidae, providing a justification for the lack of information on flounder hybrids. Analysis of genetic information and genetic mapping studies of individuals with intermediate traits between the parents support previous research suggesting that it has economic value.

## Introduction

The olive flounder, *Paralichthys olivaceus* Temminck & Schlegel, 1846 is a flatfish that is eaten in East Asia. It possesses large teeth, inhabits benthic environments, and exhibits leftward-shifted eyes (Bai and Lee 2010). In South Korea, 41,207 tons were harvested in 2017, establishing it as a valuable aquaculture species (FAO, 2018). The spotted halibut, *Verasper variegatus* Temminck & Schlegel, 1846, inhabits the East Asian coast and reaches approximately 60 cm in length. Although it offers superior nutritional value and flavor, its declining catch and low aquaculture production rate make it difficult to secure a stable supply (Ando et al. 1998; Nemoto 1999; Wada et al. 2004, 2010; Shimamura 2007). Through artificial insemination, we successfully hatched a hybrid of these two important species.

The structure and sequence of the compact, maternally inherited mitochondrial genome exhibit conservation among vertebrates, facilitating the study of phylogenetic relationships between species (Miya et al. 2003; Pardo et al. 2005). Many hybrids have been produced in attempts to create superior strains of fish for the purpose of fish cultivation. In this respect, the most interesting Pleuronectidae hybrid is the cross between *P. olivaceus*, which grows rapidly in cold water, and *V. variegatus*, which has good meat quality. Since hybrid traits are generally intermediate between those of the parents, this hybrid would be easy to grow and would have great potential. Consequently, this study aimed to determine the complete mitochondrial genome of *P. olivaceus* (♀) × *V. variegatus* (♂), enabling elucidation of its phylogenetic position.

## Materials

**Figure 1.**
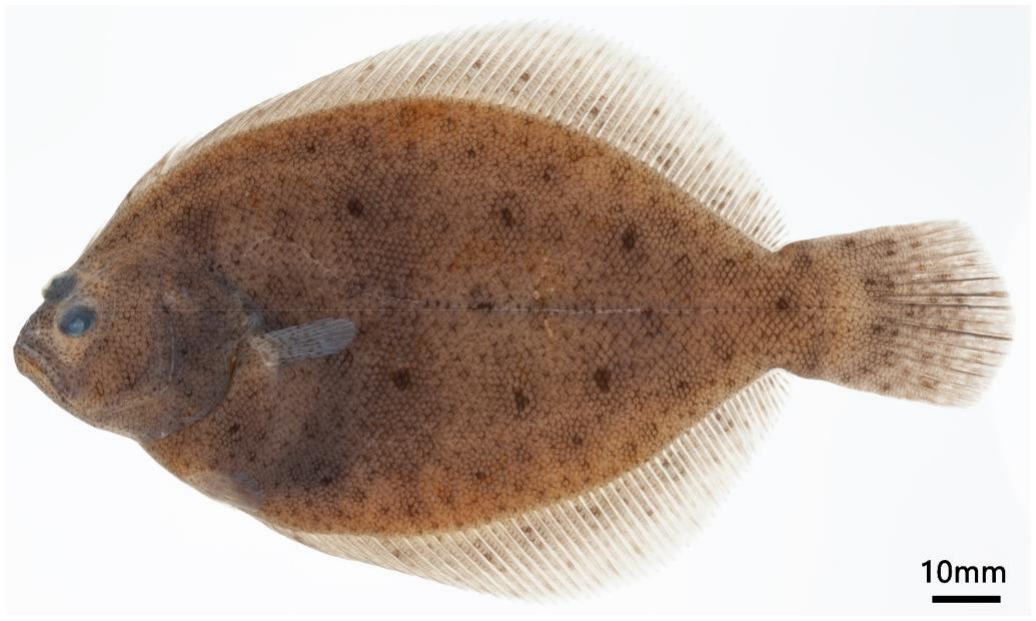
*P. Olivaceus* (♀) × *V. variegatus* (♂), SUC26325, 120 mm SL, Juvenile, Taean, Chungcheongnam, Korea, 10 April 2023. Photograph by Y.H. Kim, used with permission. The hybrid exhibits an oval body similar to its parent, with small black and white spots covering the entire body like *P. olivaceus*, and round black spots on the dorsal, pelvic, and caudal fins resembling *V. variegatus.*

In March 2023, larvae and adults of *P. olivaceus* (♀) × *V. variegatus* (♂), hatched at the Soonchunhyang University Marine Fisheries Research Institute (36°61’959"N 126°33’552"E, 236, Cheonsuman, Chungcheongnam, Republic of Korea), were immersed in 99.9% ethanol and deposited in a sample storage facility at Soonchunhyang University (Prof. I-C. Bang, incbang@sch.ac.kr), under voucher no. SUC26325 (Figure 1).

## Methods

Genomic DNA (gDNA) was extracted from the whole body of *P. olivaceus* (♀) × *V. variegatus* (♂) larvae using a HiGene^™^ Genomic DNA Prep Kit (BIOFACT, Daejeon, Republic of Korea; Cat. No. GD264-060) in accordance with the manufacturer’s protocol. A DNA library was prepared from the extracted gDNA using the MGIEasy DNA Library Prep Kit (MGI Tech, Shenzhen, China), and raw next-generation sequencing data were obtained through 150 bp paired-end reads on the MGISEQ-2000 platform (MGI Tech, Shenzhen, China). The raw data were trimmed using Cutadapt ver. 4.2 (Martin 2011), and the contig sequences were assembled using the CLC Genomics Workbench (ver. 20.04) (CLC, Aarhus, Denmark). The circular mitochondrial genome was contig sequenced using Geneious R (ver. 11.0.3) (https://www.geneious.com) (Kearse et al. 2012), and the final sequence was annotated using the MITOS web-based tool (http://mitos.bioinf.uni-leipzig.de) (Bernt et al. 2013). The complete mitogenome sequence was subsequently registered at NCBI with accession number OR353704.

The assembled raw data were aligned with ClustalW (Thompson et al. 1994) using BioEdit (ver. 7.7). Sequences were analyzed using jModelTest (ver. 2.1.10) (RRID:SCR_015244) (Darriba et al. 2012) and selected according to the Akaike Information Criterion (AIC) to place *P. olivaceus* (♀) × *V. variegatus* (♂) in a phylogenetic context. The maximum likelihood (ML) method was utilized to determine the most appropriate model (GTR+I + G).

The phylogeny was constructed using PHYML (ver. 3.0) (Guindon et al. 2010) and iTOL (ver. 6) (Letunic and Bork 2007). We referenced the phylogenetic list from a recent study of the flounder lineage (Chae et al. 2023) and incorporated three species from the maternal and paternal genera *Verasper* and *Paralichthys*. For precise intra-Osteichthyes comparisons, sturgeon (*Acipenser fulvescens*) and available Cynoglossidae NCBI sequences were adopted as out-groups. The in-group comprised all Pleuronectidae genera registered in NCBI. Both genera belong to Pleuronectiformes, and 25 species were utilized (out-group: *Acipenser fulvescens* (MT667238), *Cynoglossus roulei* (MK574671), *Cynoglossus semilaevis* (EU366230), *Cynoglossus sinicus* (JQ348998), *Paraplagusia bilineata* (NC023227), and *Paraplagusia blochii* (JQ349002); in-group: *Arnoglossus tenuis* (KP134337), *Cleisthenes herzensteini* (KT223828), *Glyptocephalus stelleri* (NC060723), *Hippoglossoides platessoides* (MN122825), *Hippoglossus hippoglossus* (AM749122), *Hippoglossus stenolepis* (AM749126), *Kareius bicoloratus* (AP002951), *Limanda aspera*(NC028281), *Microstomus achne* (OP066370), *Paralichthys olivaceus* (NC002386), *Platichthys stellatus* (EF424428), *Pleuronichthys cornutus* (JQ639071), *Pleuronichthys japonicus* (KY038655), *Pseudopleuronectes herzensteini* (ON127848), *Pseudopleuronectes yokohamae* (KT878309), *Reinhardtius hippoglossoides* (AM749130), *Verasper moseri* (NC008461), and *Verasper variegatus* (NC007939)).

## Results

**Figure 2.**
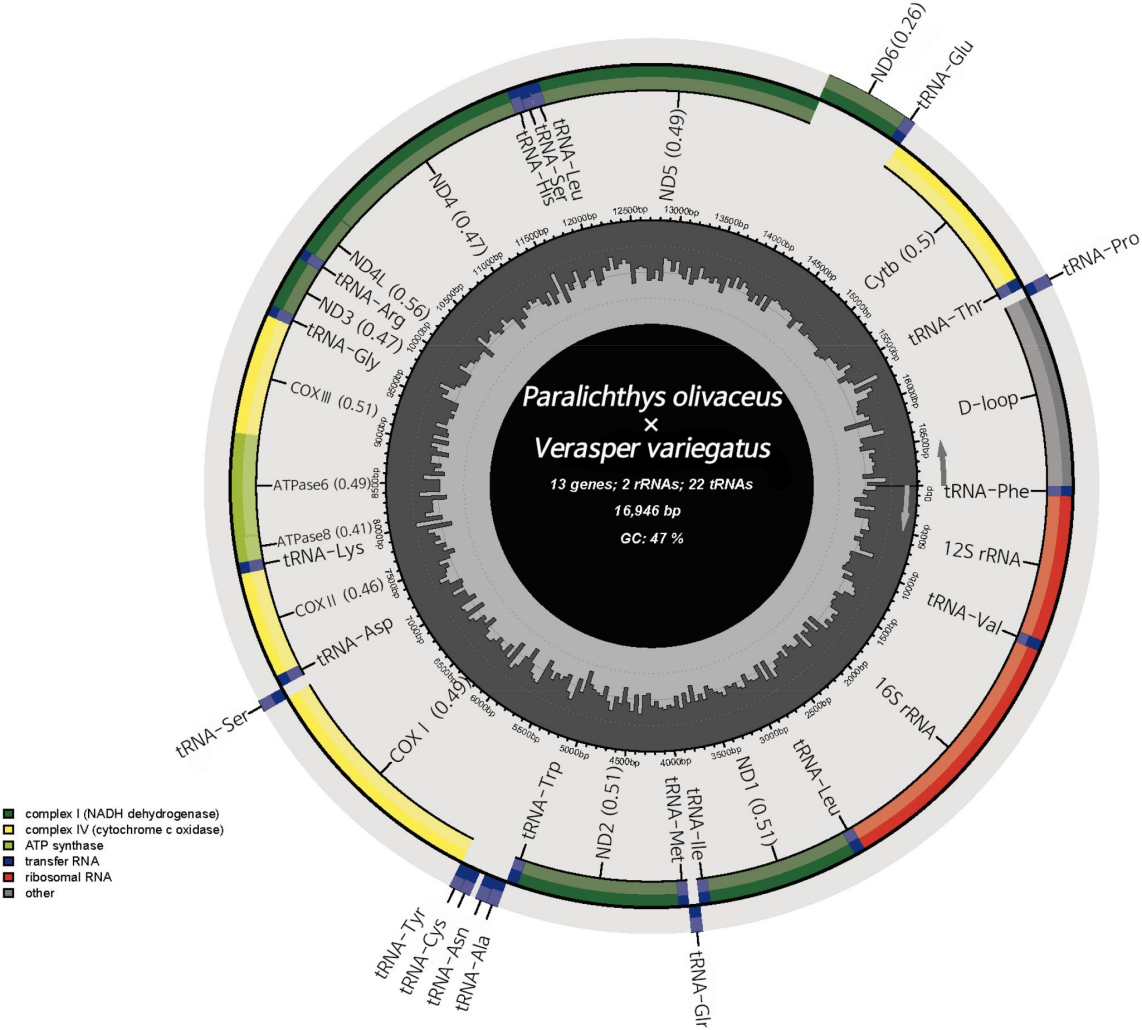
The mitochondrial genome map for *P. olivaceus* (♀) × *V. variegatus* (♂), produced on the MitoFish web server v.3.90, depicts 13 protein-coding genes, two ribosomal RNAs, 22 transfer RNAs, and a control region (GenBank acc. no.: OR353704). The mitochondrial gene order exhibits conservation among species.

The total length of the final mitogenome of *P. olivaceus* (♀) × *V. variegatus* (♂) is 16,946 bp, encompassing 13 protein-coding genes (PCGs), 22 tRNA genes, and two rRNA genes. All PCGs except *nad6* are coded on the positive strand (Figure 2). The total base composition is GC 46.99%, with adenine (A) 24.74%, cytosine (C) 30.37%, guanine (G) 16.62%, and thymine (T) 28.26%. The A + T composition is 53%, comparable to the mitogenomes of other vertebrates (Saccone et al. 1999; Zhu et al. 2013). The 13 PCGs constitute 67.44% of the total length or 11,429 bp, and the control region spans 1,256 bp. The 12S rRNA (949 bp) is located between tRNA^Phe^ and tRNA^Val^, whereas the 16S rRNA gene (1,714 bp) is located between tRNA^Val^ and tRNA^Leu^. Excluding *COX1*, 12 PCGs began with the ATG start codon; *ND1*, *COX1*, *ATP8*, *NDL4*, and *ND5* ended with a complete TAA or TAG stop codon, whereas the remaining PCGs ended with T or A.

The mitogenome of *P. olivaceus* (♀) × *V. variegatus* (♂) contains 22 tRNA genes. Of these, tRNA^Glu^, tRNA^Pro^, tRNA^Gln^, tRNA^Ala^, tRNA^Asn^, tRNA^Cys^, tRNA^Tyr^, and tRNA^Ser^ are on the negative strand; the remainder (tRNA^Thr^, tRNA^Phe^, tRNA^Val^, tRNA^Leu^, tRNA^Ile^, tRNA^Met^, tRNA^Trp^, tRNA^Asp^, tRNA^Lys^, tRNA^Gly^, tRNA^Arg^, tRNA^His^, tRNA^Ser^, and tRNA^Leu^) are on the positive strand and exhibit the typical tRNA structure.

**Figure 3.**
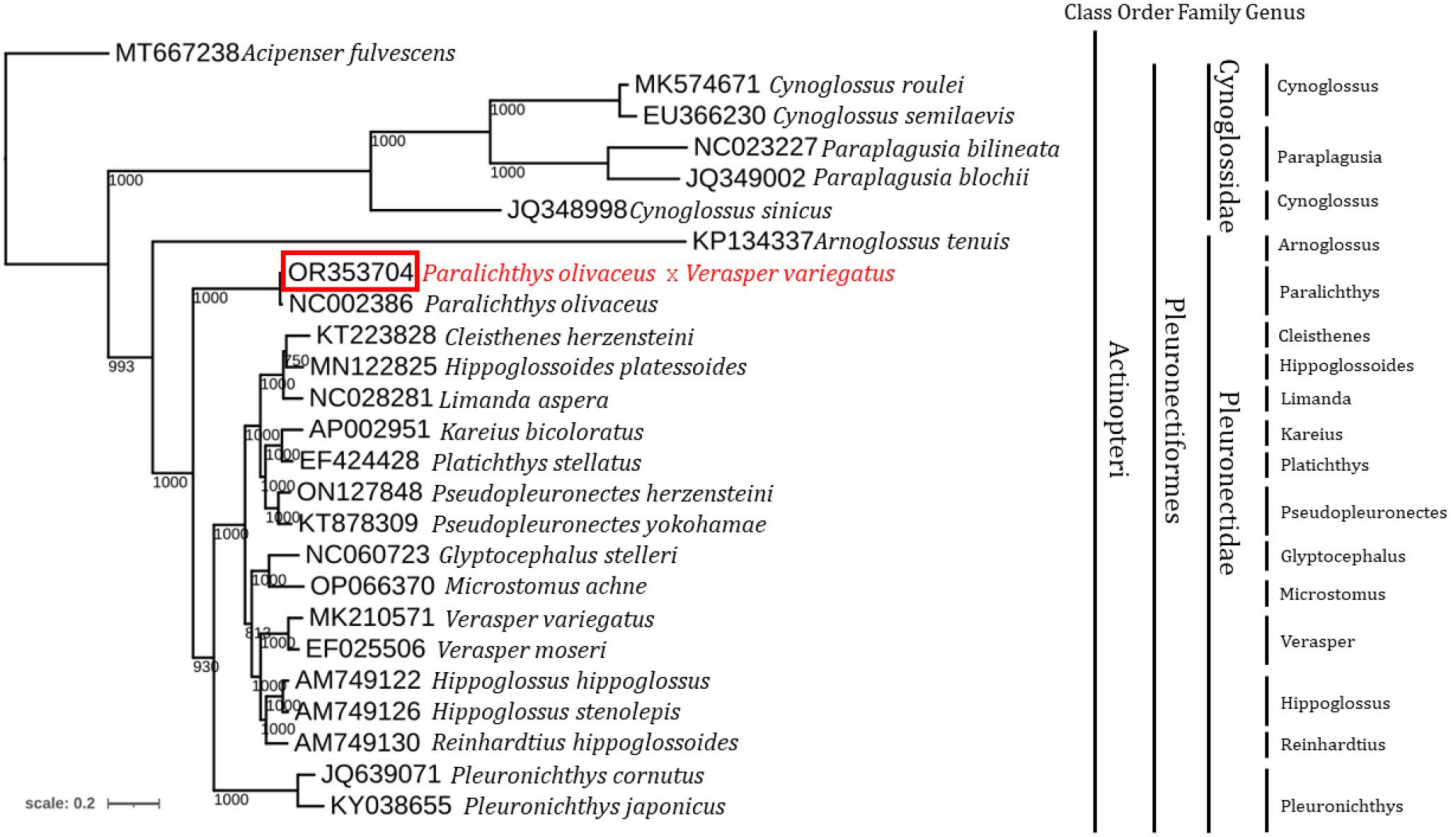
Phylogenetic tree of 13 pleuronectidae species and *P. olivaceus* (♀) × *V. variegatus* (♂) constructed using 13 mitochondrial PCGs and the maximum likelihood method. GenBank accession numbers precede the scientific names. The following sequences were used: *A. fulvescens* (MT667238.1; schroeter et al. 2020), *cynoglossus roulei* (MK574671.1; chen et al. 2019), *cynoglossus semilaevis* (EU366230.1; kong et al. 2009), *cynoglossus sinicus* (JQ348998.1; shi et al. 2012), *paraplagusia bilineata* (NC023227.1; fricke et al. 2024), *paraplagusia blochii* (JQ349002.1), *arnoglossus tenuis* (KP134337), *cleisthenes herzensteini* (KT223828.1), *glyptocephalus stelleri* (NC060723.1; xiao et al. 2010), *Hippoglossoides platessoides* (MN122825.1; margaryan et al. 2021), *Hippoglossoides hippoglossus* (AM749122.1; Mjelle a., et al. 2008), *Hippoglossoides stenolepis* (AM749126.1; vinnikov et al. 2018), *kareius bicoloratus* (AP002951.1; miya et al. 2001), *limanda aspera* (NC028281.1; vinnikov et al. 2018), *microstomus achne* (OP066370.1; chae et al. 2022), *P. olivaceus* (NC002386.1; saitoh et al. 2000), *P. stellatus* (EF424428.1; vinnikov et al. 2018), *pleuronichthys cornutus* (JQ639071.1; shi et al. 2013), *pleuronichthys japonicus* (KY038655.1; song et al. 2017), *pseudopleuronectes herzensteini* (ON127848.1; chae et al. 2022), *pseudopleuronectes yokohamae* (KT878309.1; liu et al. 2017), *reinhardtius hippoglossoides* (AM749130.1; Mjelle a., et al. 2008), *verasper moseri*, and *V. variegatus* (NC008461.1, NC007939.1).

Each species was classified as belonging to the family Pleuronectidae, family Cynoglossidae, or outgroup (*A. fulvescens*). *P. olivaceus* (♀) × *V. variegatus* (♂) clustered with *P. olivaceus* in Pleuronectidae and was analyzed as a distinct node in the phylogeny (Figure 3).

## Discussion and conclusion

This study presents the first determination of the complete mitochondrial genome of a second-generation hybrid obtained through artificial crosses between *P. olivaceus* and *V. variegatus*, revealing its genetic characteristics. The mitogenome composition aligns with that of a typical vertebrate (Pereira 2000). In an analysis of several Pleuronectidae family genera, *P. olivaceus* (♀) × *V. variegatus* (♂) clustered with the maternal genus *Paralichthys* with high bootstrap values (1,000 BP). This observation aligns with other studies (Debes et al. 2013; Hou and Liu 2018; Li et al. 2018), suggesting that hybrids are significantly influenced by the maternal lineage. The examined hybrid likely possesses substantial economic value, based on previous research indicating growth rates similar to those of the olive flounder (Kim et al. 1995). In conclusion, the whole-genome study of this species presents a molecular phylogeny favorable for aquaculture cultivar development, and analysis of its genetic information in Pleuronectidae provides ideal material for genetic mapping and genomic studies of hybrids.

## Supplementary Material

S1_OR353704 mapping depth.jpg

S2_Electropherograms of the rag1 gene demonstrate clear evidence of hybridization between Paralichthys olivaceus and Verasper variegatus_Double peaks are indicated by red arrows.jpg

## Data Availability

The genome sequence data that support the study findings are openly accessible in NCBI GenBank at https://www.ncbi.nlm.nih.gov/ under accession no. OR353704. The associated BioProject, SRA, and Bio-Sample numbers are PRJNA1049058, SRS19795251, and SAMN38681716, respectively.

## Additional information

The English in this document has been checked by at least two professional editors, both native speakers of English. For a certificate, please see:

http://www.textcheck.com/certificate/5a4R0C
