# Supplementary figures and images for: Complete mitochondrial genome of the hybrid flounder Paralichthys olivaceus (♀) × Verasper variegatus (♂)
Source: Mitochondrial DNA B Resour. 2025 May 8;10(6):425–9. doi: 10.1080/23802359.2025.2498746 (PMC12064119; doi:10.1080/23802359.2025.2498746)

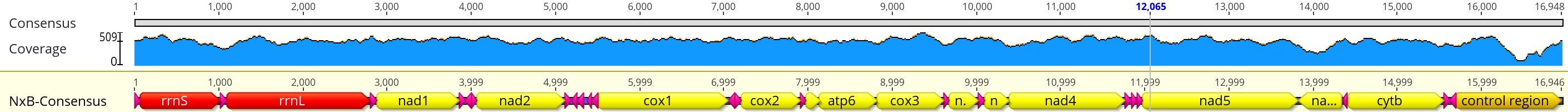

Supplement: S1_OR353704 mapping depth.jpg [file TMDN_A_2498746_SM7387.jpg]

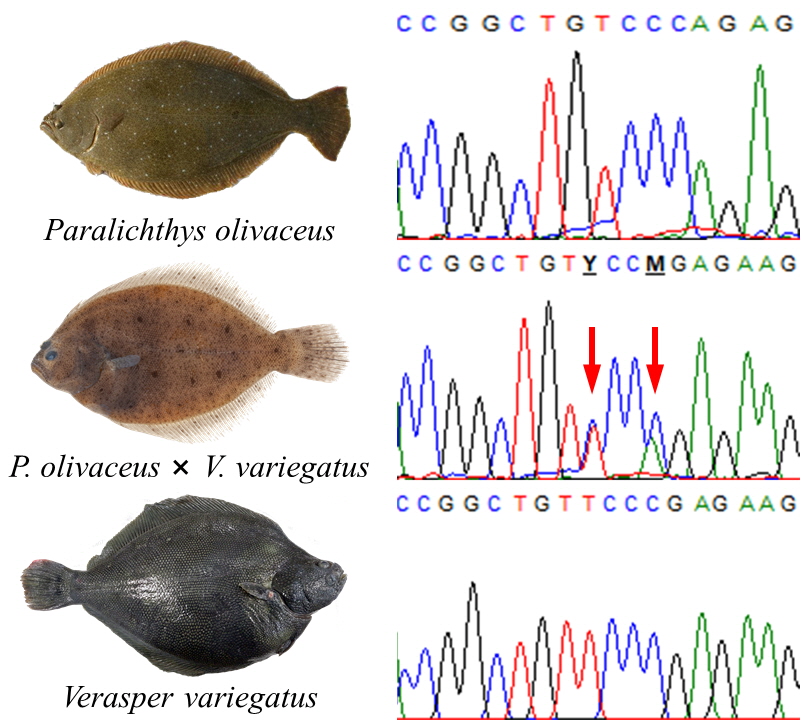

Supplement: S2_Electropherograms of the rag1 gene demonstrate clear evidence of hybridization between Paralichthys olivaceus and Verasper variegatus_Double peaks are indicated by red arrows.jpg [file TMDN_A_2498746_SM7386.jpg]
